# Comparative trial of the effects of continuous locomotion training provided at pharmacies: a pilot study

**DOI:** 10.1186/s40780-020-00182-8

**Published:** 2020-11-23

**Authors:** Chigusa Kikuchi, Kyoko Yamaguchi, Masayo Kojima, Haruyuki Asai, Rika Nakao, Yoshifusa Otake, Junya Nagata, Shinpei Matsunami, Asako Horiba, Tadashi Suzuki

**Affiliations:** 1 Department of Clinical Pharmacy, Graduate School of Pharmaceutical Sciences, Nagoya City University, 3-1 Tanabe-dori, Mizuho-ku, Nagoya, 467-8603 Japan; 2 Educational Research Centre for Clinical Pharmacy, Faculty of Pharmaceutical Sciences, Nagoya City University, 3-1 Tanabe-dori, Mizuho-ku, Nagoya, 467-8603 Japan; 3 Department of Medical Education, Graduate School of Medicine, Nagoya City University, 1 Kawasumi, Mizuho-ku, Nagoya, 467-8603 Japan; 4 Asai Pharmacy Tachibana Branch, 6-24-1 Tachibana-cho, Tsushima, Aichi 496-0038 Japan; 5 Haruka Pharmacy Sakoh, 2-5-11 Sakoh, Nishi-ku, Nagoya, 451-0052 Japan; 6 Yamazaki Pharmacy Kamijima Branch, 2-11-10 Kamijima, Naka-ku, Hamamatsu, 433-8122 Japan; 7 Kyowa Pharmacy Kariya Ekimae Branch, 1-58-102 Minamisakura-cho, Kariya, Aichi 448-0841 Japan; 8 Shoeido Pharmacy, 1-48-101 Yutaka, Minami-ku, Nagoya, 457-0863 Japan; 9 Olive Pharmacy Asada Branch, 302 Nishimaeda, Asada-cho, Nisshin, Aichi 470-0124 Japan

**Keywords:** Pharmacy, Elderly, Locomotive syndrome, Locomotion training, Community, Health support

## Abstract

**Background:**

While the world’s population is growing older, healthy life expectancy is not increasing. The Japanese Orthopedic Association proposed the concept of ‘locomotive syndrome,’ manifested as a decline in mobility functions, and introduced a short test battery for assessing the risk of this syndrome. The test battery includes the ‘stand-up test,’ ‘two-step test,’ and ‘25-question Geriatric Locomotive Function Scale’ (25-question GLFS). The purpose of locomotion training is to improve and sustain standing and gait functions. However, the place where locomotion training can be provided and followed up has not been decided upon. Therefore, a study was conducted to explore the effect of locomotive syndrome improvement by continuous locomotion training provided at community pharmacies. The objective of this study was to evaluate the effect of pharmacists’ instructions and follow-up on the compliance and effectiveness of locomotion training.

**Methods:**

The inclusion criteria were 1) age ≥ 65 years and 2) decline in mobility functions. Guidance on how to perform locomotion training was provided by a pharmacist at the pharmacy. The participants performed locomotion training at home. They were tested and instructed at the pharmacy once a month for 3 months. The main outcome measures were test battery results and the percentage of number of days participants who were able to do the training at home.

**Results:**

Eleven participants were analysed. The minimum implementation percentage was 78%. Improvements were observed in 25-question GLFS, muscle strength, and standing time on one leg. Three participants no longer showed a noticeable decline in mobility function.

**Conclusion:**

Continuous locomotion training provided at pharmacies could contribute to locomotive syndrome prevention.

**Trial registration:**

This study was registered with the University hospital Medical Information Network Clinical Trials Registry (UMIN-CTR; identification No. UMIN000027963. Registered 28 June 2017).

## Background

The world’s elderly population is growing at an unprecedented rate. This phenomenon is considerably more pronounced in the Japanese population. Japan continues to lead the percentage of the population older than 65 years (28%) [1]. Furthermore, the average life span of Japanese people has considerably increased in recent years. However, healthy life expectancy is shorter than overall life expectancy. The timespan from healthy life to death is approximately 10 years [2]. In Japan, the number of people who require long-term care is on the rise. In 2011, 3.783 million people needed long-term care, whereas in 2016, the number increased to 4.459 million [2]. To address this issue, it is vital to take measures that can extend the healthy life expectancy of people.

Amongst the various reasons for long-term care, the locomotor disorder is a major one, accounting for 22.7% in 2016 [2]. In 2007, the Japanese Orthopedic Association proposed the concept of ‘locomotive syndrome’ (Locomo), wherein a decline in mobility functions such as sit-to-stand or gait decline, was observed due to an impairment of locomotive organs [3]. To improve and sustain standing and gait functions, a method of locomotion training, called ‘Locotra,’ has been recommended [3]. This training consists of exercises such as standing on one leg with eyes open and squatting. The beneficial effects of these exercises have been reported by other researchers [4, 5]. Although Locotra is safe and feasible to practice at home, the location where instructions should be provided, and subsequent follow-up has not been decided upon.

Considering the rising medical costs in Japan, it is important to prevent lifestyle-related diseases. To address this issue, the health support pharmacy system was introduced in 2016 in Japan [6–10]. The role of a pharmacy is to help the community to improve its health. However, the new services leave the contents up to each pharmacy. Pharmacies have the following three advantages: a pharmacist and a medical specialist are always present at the pharmacy, stores are present at regular intervals, and people can easily access pharmacies when compared to hospitals. We considered the possibility of establishing a new pharmacy health support by exploiting these advantages. Hence, it is the responsibility of the pharmacists to decide which service is needed and how to provide the new service.

The purpose of this study was to examine the effect of pharmacists’ instructions and follow-up of Locotra in their community, as the health support pharmacy system. We evaluated pharmacist instructions and conducted a follow-up of Locotra in citizens of the community and tested the effects of Locotra at pharmacies.

## Methods

### Training pharmacists

The study was conducted at pharmacies in Aichi Prefecture and western Shizuoka Prefecture that consented to participate in this study. Before conducting the study, the pharmacists were trained to acquire sufficient knowledge and skills about Locomo and Locotra from the Care Prevention Network Association, an organization that promotes Locotra. The contents of the training were as follows: epidemiology, pathophysiology, Locomo degree judgement method, and locomotion training instruction method.

### Participants

Locomo clinical decision limits were established in two stages. The beginning of the decline in mobility functions was categorized as stage 1, and a progression in mobility function decline was categorized as stage 2 [4]. The participants of this study were those with Locomo stage 1. Pharmacy visitors were recruited as participants for this study. A pharmacy visitor was any local resident who visited a pharmacy (with or without a prescription) to buy medicine, for consultation, or for any other reason. The inclusion criteria were as follows: (1) Japanese, aged ≥65 years, and (2) inability to stand on one leg at the height of 40 cm. The exclusion criteria were as follows: (1) inability to exercise, (2) requirement of long-term care, (3) diagnosis of mental illness, (4) exercise guidance at specialized facilities, such as gymnasiums, (5) Locomo stage 2, (6) no visit to the pharmacy after the observation period, (7) extremely limited range of joint motion, (8) pain, swelling, inflammation, damage, paralysis, and skin disorders in the lower limbs, (9) hypersensitivity to measuring instrument materials, and (10) judged by the investigator to be ineligible.

All procedures followed were in accordance with the ethical standards of author’s institution Clinical Research Review Board and the Helsinki Declaration of 1964 and later versions. Informed consent was obtained from all participants before inclusion in the study. This study was registered with the University hospital Medical Information Network Clinical Trials Registry.

### Observation period

The observation period was 28 ± 7 days from the pre-intervention assessment date. During the observation period, we examined the registered participants with respect to the number of visits to the pharmacy, guidance contents about health at the pharmacy, and their health status.

Clinical decision limits were established in two stages (Table 1). The following criteria were used to indicate the beginning of a decline in mobility functions and the participant was categorized as stage 1 if any of the following three conditions were met: stand-up test score ≥ 3 and < 5, two-step test ≥1.1 and < 1.3, and 25-question GLFS score ≥ 7 and < 16. The following criteria were used to indicate a progression in mobility function decline, and the subject was categorized as stage 2 if any of the three conditions were met: stand-up test score < 3, two-step test < 1.1, and 25-question GLFS score ≥ 16 [3].

**Table 1:** Locomotive syndrome clinical decision limits

	Stage 1	Stage 2
Stand-up test score	≥ 3 and < 5	< 3
Two-step test score	≥ 1.1 and < 1.3	< 1.1
25-question GLFS	≥ 7 and < 16	≥ 17

Stand-up test score is expressed as the height of the lowest stool from which the subject was able to stand-up: all failed = 0, 40 cm with both legs = 1, 30 cm with both legs = 2, 20 cm with both legs = 3, 10 cm with both legs = 4, 40 cm with a single-leg = 5, 30 cm with a single-leg = 6, 20 cm with a single-leg = 7, and 10 cm with a single-leg = 8. Two-step test score was calculated by normalising the maximal length of two steps taken by the participant by his/her height; *25-question GLFS* 25-question geriatric locomotive function scale

The leg muscle strength was evaluated using Locomoscan (Alcare Co., Ltd., Tokyo, Japan), an instrument that measures quadriceps strength [11, 12]. Standing time on one leg with the eyes open was measured for a maximum of 60 s, during which one leg could be raised by approximately 5 cm without losing balance [13]. The SF-8™ Health Survey (Standard, Japanese version; iHope International Co., Ltd., Kyoto, Japan) is a short, comprehensive, and versatile survey that measures health [14–16]. This survey was also employed in this study, according to the manual instruction [14]. Besides, we examined the following characteristics of the participants: age, sex, family unit, disease, participation in society, number of days spent outdoors, sleeping on a futon (Japanese style of bedding directly on the floor without a bed), or bed, and conducting household activities.

### Intervention method

After the completion of the above tests, the participants were provided instructions regarding Locotra. The two exercises included in Locotra were single-leg standing with the eyes open and squatting. The former involved the participants standing on one leg with their eyes open for 1 min. One set comprised the exercises performed once for each leg at a time. The participants were instructed to perform three sets every day [3]. For squatting, the participants slowly moved the torso down from the standing position, similar to stand-sit movement. One set comprised 5–6 slow squats and three sets had to be performed every day [3]. Thereafter, the participants visited the pharmacy once a month, where the stand-up test, two-step test, 25-question GLFS, measurement of muscle strength, SF-8™ Health Survey (Standard, Japanese version), and measurement of standing time on one leg with open eyes were carried out. Moreover, we checked the implementation status of Locotra, and checked that the participant was performing Locotra following the correct procedure. The pharmacist consultation was at the discretion of the pharmacist. When the participant visited the pharmacy, the pharmacist made an appointment for the next available day. The study was completed on the third visit.

### Statistical analysis

Changes in measurements due to the intervention were compared to the baseline and analysed with Wilcoxon’s signed-rank test. A correlation analysis between changes in parameters and frequency of Locotra was conducted with Spearman’s rank correlation coefficient. A statistical significance threshold of 5% was used (*p* < 0.05). All statistical analyses were carried out using IBM SPSS Statistics for Windows, Version 26 (IBM Corp., Armonk, NY, USA).

## Results

Informed consent was obtained from 16 people, of which 12 (who met the selection criteria) were registered in the study. Twelve participants completed the study, but only 11 were included in the analysis because one participant deviated from the protocol (the first monthly inspection interval was 27 days, which was shorter than the default 28 days or more). The baseline characteristics of the participants are presented in Table 2.

**Table 2:** Baseline Characteristics of Participants

Characteristic	Participants *(n* = 11)
Median age, years (range)	74 (68–82)
Sex	
Male / Female	4 / 7
Family unit	
Single / Couple / Other	3 / 2 / 6
Disease	
Hypertension / Diabetes / Hernia / Prostatic hyperplasia / None	5 / 2 / 1 / 1 / 2
Social engagement	
Yes / No	9 / 2
Outdoor activity, median number of days in a month (range)	30 (12–30)
Sleeping on	
Futon / Bed	7 / 4
Household activities	
Yes / No	11 / 0

The implementation rates of Locotra are presented in Table 3. The results of the single-leg standing test (with the eyes open) and squatting test were similar. At least once a day, the lowest percentage of implementation was 78% throughout the study. Similar results were observed for squats.

**Table 3:** Implementation Percentage of Locomotion Training

	1st month *n* = 11	2nd month *n* = 11	3rd month *n* = 11	Total *n* = 11
	Median (range) %	Median (range) %	Median (range) %	Median (range) %
Frequency of single-leg standing with the eyes open				
None	3 (0–38)	0 (0–19)	0 (0–41)	7 (0–22)
One time a day	9 (0–74)	7 (0–82)	7 (0–93)	10 (0–82)
Two times a day	24 (0–84)	46 (0–89)	54 (0–82)	38 (0–81)
Three times a day	43 (0–100)	39 (0–100)	29 (0–100)	46 (0–100)
At least one time a day	97 (63–100)	100 (81–100)	100 (59–100)	93 (78–100)
Frequency of squats				
None	3 (0–38)	0 (0–19)	0 (0–41)	7 (0–22)
One time a day	6 (0–74)	5 (0–82)	7 (0–93)	10 (0–82)
Two times a day	24 (0–84)	46 (0–89)	54 (0–82)	38 (0–81)
Three times a day	43 (0–100)	39 (0–100)	29 (0–100)	46 (0–100)
At least one time a day	97 (63–100)	100 (81–100)	100 (59–100)	93 (78–100)

Table 4 presents the changes in the locomotive syndrome-related test results. After 3 months, the score of 25-question GLFS decreased significantly. In addition, leg muscle strength and standing time on one leg, with the eyes open increased. Consequently, three participants were no longer in Locomo stage 1. Furthermore, to verify the correlation between these values and the implementation rate, we performed Spearman’s rank correlation coefficient. As shown in Table 5, the 25-question GLFS and left leg muscle strength correlated with the implementation percentage of days for single-leg standing with the eyes open. However, no correlation was observed with the number of times nor days of locomotive training.

**Table 4:** Changes in the Locomotive Syndrome-Related Test

	Baseline	1 month	2 months	3 months	Baseline vs. 3 months *p-*value
Stand-up test (*n* = 11) Median (range)	3 (3–5)	3 (3–5)	3 (3–5)	4 (3–5)	0.083
Two-step test (*n* = 11) Median (range)	1.4 (1.1–1.8)	1.4 (1.2–1.7)	1.4 (1.2–1.7)	1.3 (1.2–1.7)	0.739
25-question GLFS (*n* = 11) Median (range)	6 (1–12)	3 (0–12)	2 (0–8)	1 (0–7)	0.014
No locomotive syndrome (n)	0	1	2	3	–
Leg muscle strength (*n* = 8)					
Right leg [Newton]	322.5	389	403	391	0.025
Median (range)	(187–429)	(173–471)	(197–493)	(239–521)	
Left leg [Newton]	361	363	424.5	399	0.025
Median (range)	(238–519)	(216–508)	(259–559)	(257–584)	
Standing time on one leg with the eyes open (*n* = 11)					
Right leg [s] Median (range)	40 (12–60)	58 (10–60)	60 (7–60)	47 (20–60)	0.044
Left leg [s] Median (range)	40 (3–60)	60 (20–60)	60 (20–60)	60 (15–60)	0.021
SF-8™ Health Survey (*n* = 11)					
PCS Median (range)	53 (42–56)	52 (44–59)	5 (42–58)	54 (46–58)	0.374
MCS Median (range)	52 (43–57)	54 (45–57)	54 (42–56)	52 (46–56)	0.859

*25-question GLFS* 25-question geriatric locomotive function scale, *PCS* physical component summary, *MCS* mental component summary

**Table 5:** Correlations between Changes in Parameters and Frequency of Locomotion Training

	Single-leg standing with the eyes open			Squats		
	Number of times	Number of days	Implementation percentage of days	Number of times	Number of days	Implementation percentage of days
25-question GLFS (*n* = 11)	−0.235	−0.457	−0.640*	−0.235	−0.457	−0.640*
Leg muscle strength (*n* = 8)						
Right leg	−0.167	− 0.390	− 0.386	−0.167	− 0.390	−0.386
Left leg	−0.024	0.561	0.747*	−0.024	0.561	0.747*
Standing time on one leg with the eyes open (*n* = 11)						
Right leg	−0.187	−0.580	− 0.280	−0.187	− 0.580	−0.280
Left leg	0.193	0.413	0.451	0.193	0.413	0.451

Values are expressed as Spearman’s rank correlation coefficient (*ρ*-value) for nonparametric variables. *p < 0.05. *25-question GLFS* 25-question geriatric locomotive function scale

An increase in the number of visits to the pharmacy was reported in four out of eleven participants. At the start of the study, pharmacists provided consultation to one participant. However, by the end of the study, nine participants received consultation by the pharmacists. While consultation regarding Locomo was most frequently provided (*n* = 10), consultations regarding cold, skin, blood glucose, stress, light-headedness, health foods, and family health (each, *n* = 1) were also provided. The purchase of over the counter drugs was reported in two participants. Information on the health status, ascertained by the pharmacist, was reported for all 11 participants, while information on body pain, medical history, and weight fluctuation was obtained from 11, 5, and 2 participants, respectively.

## Discussion

In this study, we demonstrated that a pharmacy could act as a health support system and provide instructions and follow-up to the community regarding Locotra. Several studies have documented the effectiveness of Locotra. For example, three locomotion training sessions were conducted for elderly home-dwelling people, and the results for the 25-question GLFS, stand-up test, and two-step test improved for those who attended all three sessions compared with those who were absent for more than one session [4]. In another study, it was reported that the results of the one leg standing test and sit-to-stand test were improved by home-visit locomotion training, with telephone support, for elderly people who were not attending any preventive-care program [5]. However, a suitable location for the dissemination of Locotra instructions and follow-up has not been established. In this study, we successfully demonstrated that a pharmacy is a suitable place to provide instructions for Locotra and subsequent follow-up.

Adherence to Locotra is usually difficult. It has been reported that without intervention, 40% of people do not use Locotra 2 years after the administration of the training [17]. However, following-up with the participants via telephone once every 2 weeks reduced the number of people who did not perform Locotra to 14.9% [18]. In this study, the elderly participants were instructed at the pharmacies and followed up regarding Locotra once a month for 3 months. Results showed that all 11 individuals were able to continue Locotra until the end of the observation period, with a Locotra implementation percentage of at least 78%. In addition, it was noted that the elderly participants could continue Locotra safely during the study. Consequently, the results of the 25-question GLFS, straight leg raising test, and standing time on one leg improved overtime. These findings were consistent with those of a previous study [4]. It should be noted that the effect of Locotra was positively correlated with the implementation percentage of days of Locotra and not the number of Locotra exercises. Previous studies have indicated no change in the muscle function with three sets and one set of the same exercise per day [19]. It is important to educate the participants to continue practicing Locotra without trying too hard. The pharmacist can play the role of an escort runner to prevent Locomo by regularly measuring the Locomo degree and providing guidance and follow-up to Locotra for the elderly people.

The effects of other health support services have also been reported [6–10]. In the UK, Healthy Living Pharmacies has been providing these services since 2009 [20]. Furthermore, Locotra has increased the number of consultations at pharmacies. Hence, Locotra improves communication between the community and pharmacists. Our results suggest that instruction and follow-up of Locotra at pharmacies might be a novel effective service as a part of a health support pharmacy system.

Notably, this study had some limitations. The number of participants in this study was small. In addition, this study was performed without a control group. The differences in the teaching ability of pharmacists could not be analyzed. Nutrition and diet of the participants was not considered. We did not analyze the differences with respect to gender, age group, and family structure of the participants. Thus, in future, it is necessary that the effects of continuous locomotion training should be investigated based on the characteristics of the participants. Furthermore, the results require validation in a larger comparative study. Nevertheless, the results from this study clearly indicated that Locotra may be applied at pharmacies, and thereby extend the healthy life expectancy of citizens.

## Conclusions

Pharmacists provided locomotive training instructions to elderly people at the pharmacy. The elderly were able to continue locomotive training for 3 months. Improvements were observed in 25-question GLFS, muscle strength, and standing time on one leg. Thus, continuous locomotion training provided at the pharmacies could contribute to the prevention of locomotive syndrome.

## Data Availability

The datasets used and/or analyzed during the current study are available from the corresponding author on reasonable request.

## Abbreviations

Locomo: Locomotive syndrome
25-question GLFS: 25-question Geriatric Locomotive Function Scale
UMIN-CTR: University hospital Medical Information Network Clinical Trials Registry
